# Microvesicles and chemokines in tumor microenvironment: mediators of intercellular communications in tumor progression

**DOI:** 10.1186/s12943-019-0973-7

**Published:** 2019-03-30

**Authors:** Xiaojie Bian, Yu-Tian Xiao, Tianqi Wu, Mengfei Yao, Leilei Du, Shancheng Ren, Jianhua Wang

**Affiliations:** 1 Cancer institute, Fudan University Shanghai Cancer Center, Fudan University, Shanghai, China; 2 Department of Urology, Shanghai Changhai Hospital, Second Military Medical University, Shanghai, China; 3 School of Medicine, Anhui University of Science & Technology, Huainan, Anhui China

**Keywords:** Microvesicles, Chemokines, Tumor microenvironment, Tumor progression

## Abstract

Increasing evidence indicates that the ability of cancer cells to convey biological information to recipient cells within the tumor microenvironment (TME) is crucial for tumor progression. Microvesicles (MVs) are heterogenous vesicles formed by budding of the cellular membrane, which are secreted in larger amounts by cancer cells than normal cells. Recently, several reports have also disclosed that MVs function as important mediators of intercellular communication between cancerous and stromal cells within the TME, orchestrating complex pathophysiological processes. Chemokines are a family of small inflammatory cytokines that are able to induce chemotaxis in responsive cells. MVs which selective incorporate chemokines as their molecular cargos may play important regulatory roles in oncogenic processes including tumor proliferation, apoptosis, angiogenesis, metastasis, chemoresistance and immunomodulation, et al. Therefore, it is important to explore the association of MVs and chemokines in TME, identify the potential prognostic marker of tumor, and develop more effective treatment strategies. Here we review the relevant literature regarding the role of MVs and chemokines in TME.

## Background

Cells generate extracellular vesicles (EVs) which are small lipid membrane-enclosed particles and function as pivotal mediators of intercellular communication by transporting biological information between cells and their microenvironment [1]. Many cell types, ranging from embryonic stem (ES) cells [2, 3] to highly malignant cancer cells [4–6], are capable of releasing different classes of EVs. In terms of pathophysiological processes, EVs have been established as important players contributing to the development and progression of cancer, and are of relevance to diseases of various sorts [7–10], including autoimmune, inflammatory, cardiovascular, hematologic, and other diseases. Two main types of EVs have been described as exosomes and microvesicles (MVs) [1, 11]. In addition, recent data have demonstrated the existence of additional varieties of EVs, which may differ in size, biogenesis, and molecular cargo profiles [12].

Chemokines are a superfamily of small, chemoattractant cytokines that bind to and activate a family of the G-protein-coupled cell-surface receptors [13]. In cancer, chemokines and their receptors are important regulators for cell trafficking in and out of the tumor microenvironment (TME) [14]. In the TME, cancer cells and surrounding non-cancerous cells constantly exchange information via gap junctions, tunneling nanotubes and effector molecules. Membrane-enclosed EVs is one of the important cargos to ensure coordinated release of multiple molecules by packaging them together [15].

### The biogenesis of MVs and chemokines

**Fig. 1:**
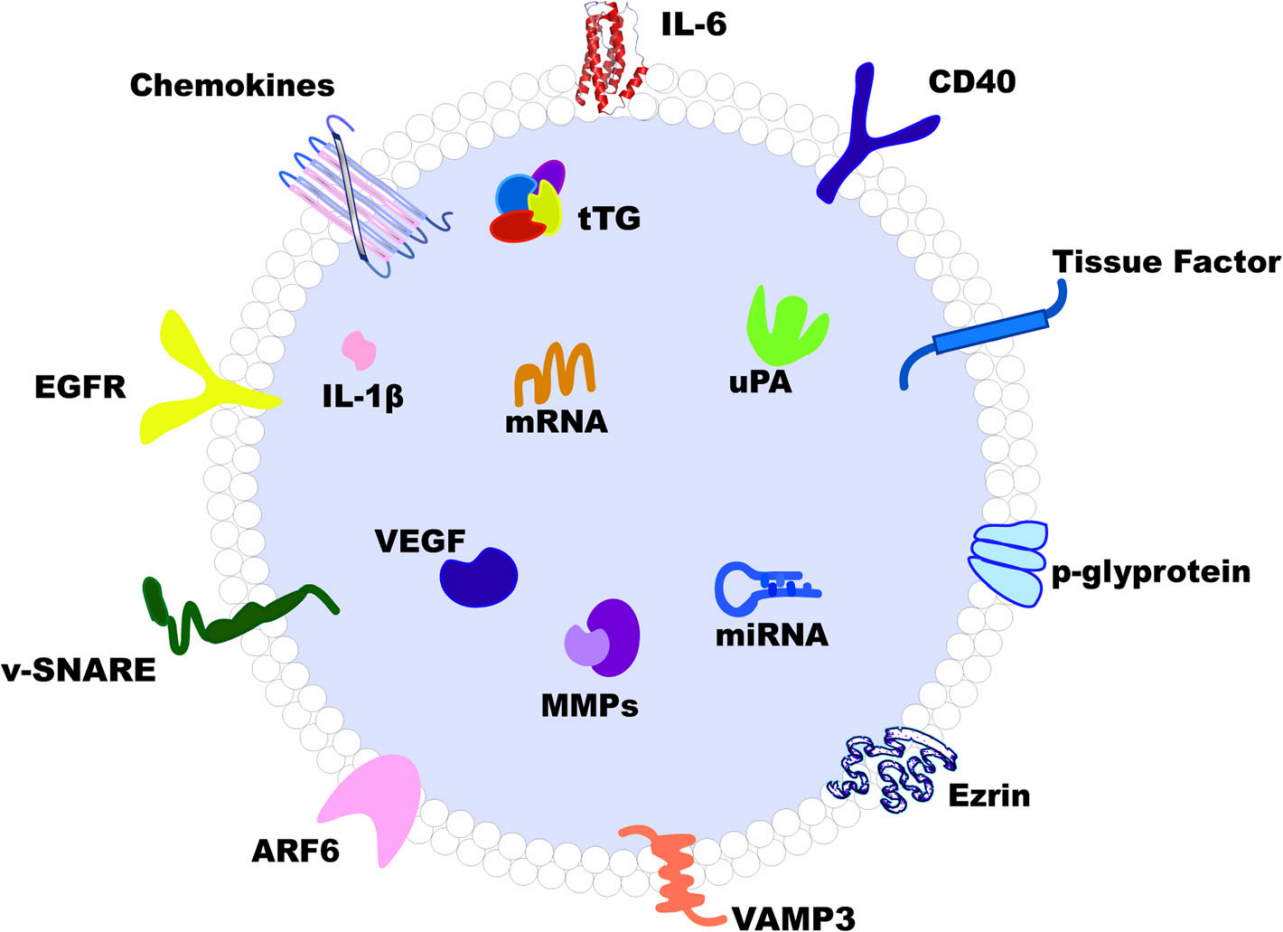
Schematic structure model of microvesicle. ARF6: ADP-ribosylation factor 6, CD40: cluster of differentiation 40, EGFR: epidermal growth factor receptor, IL-1β: interleukin-1β, IL-6: interleukin-6, MMP: matrix metalloproteinase, tTG: tissue transglutaminase, uPA: urokinase plasminogen activator, VAMP-3: vesicle-associated membrane protein 3, VEGF: vascular epithelium growth factor, v-SNARE: vesicular soluble N-ethylmaleimide-sensitive factor attachment protein receptor

MVs, also commonly referred to as ectosomes or microparticles, are significantly larger in size than exosomes (100–1000 μm in diameter) [6, 16, 17] (Fig. 1). Unlike exosomes, the release of MVs typically involves centrifugal budding in specific areas of the plasma membrane [18]. Upon the release of Ca2^+^ from the endoplasmic reticulum, the plasma membrane undergoes molecular rearrangement at the sites where MVs originate, followed by direct shedding and instantaneous release of the vesicle into the intercellular space [10, 19]. MVs contain parental intracellular information and inherit partial cell membrane markers from which they are generated. Several proteins have been proposed MVs-specific, including selectins, integrins, CD40, matrix metalloproteinase (MMP), phosphatidylserine (PS), ADP-ribosylation factor 6 (ARF6) and Rho family members [11, 20]. Different types of MVs can form in various physiological and pathological conditions. Apoptotic blebs, for instance, are microvesicles released by cells upon the trigger of the cellular collapse that results in fragmentation of nucleus, increase in permeability of the plasma membrane, and externalization of PS [21]. During apoptosis, cellular components enclosed by apoptotic blebs are actively transferred from the apoptotic cell into peripheral vesicles [22]. Another example is the recently identified cancer-derived EV population, often termed as “large oncosome”, which is considerably larger than most known EV types characterized to date [11]. Biogenesis of large oncosomes is particularly notable in tumor cells with an amoeboid phenotype, which tend to be more aggressive. Similar to MVs, this EV population might originate directly from plasma membrane budding and, similar to MVs, these particles express ARF6 [23, 24].

**Fig. 2:**
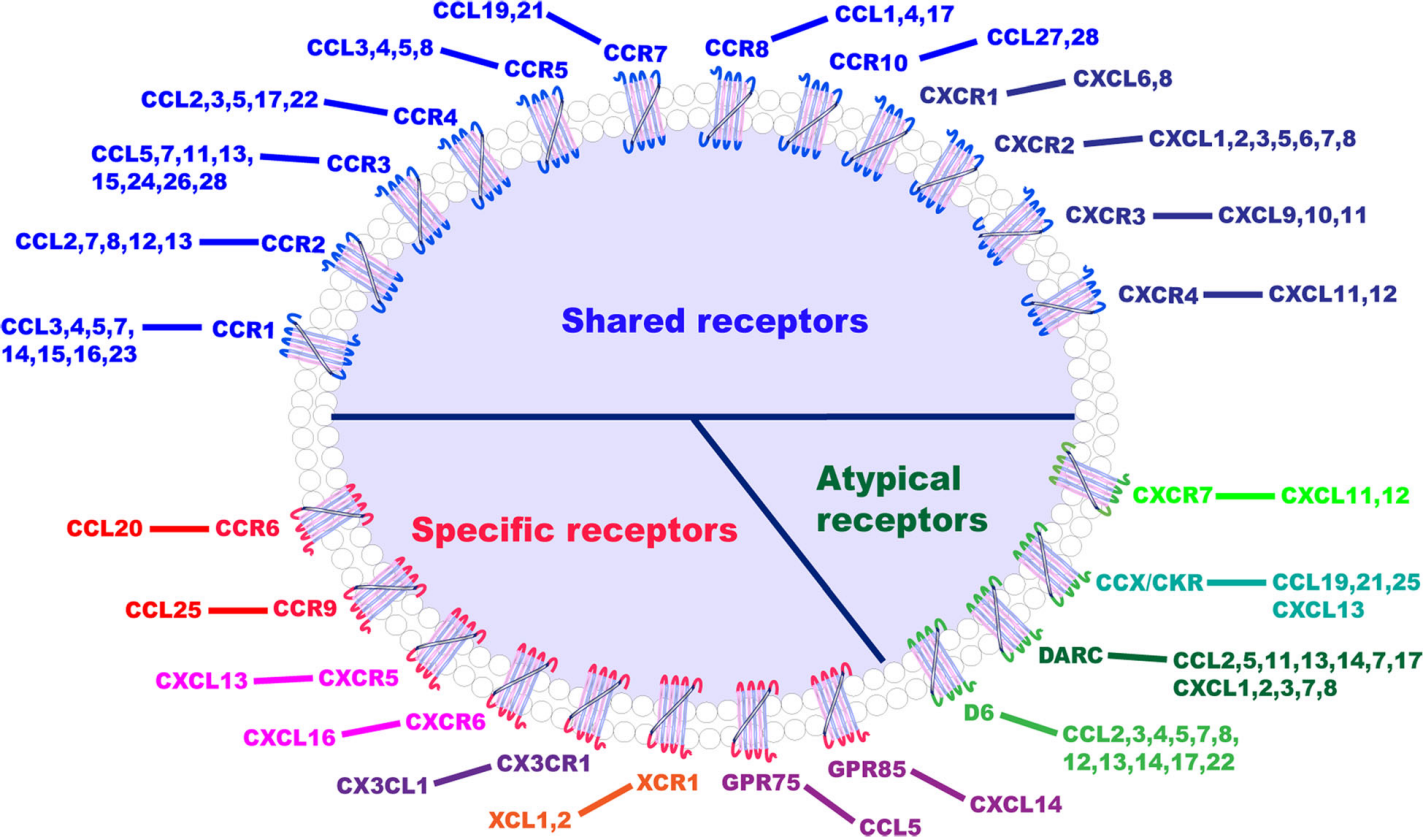
Components of the chemokine systems. The majority of chemokines can bind a series of cognate receptors, and a single receptor can bind multiple chemokines, as exhibited in this paradigm for most CC (blue) and CXC (dark blue) chemokines. Atypical receptors (green) can also interact with numerous chemokines. On the contrary, a minority of receptors (red) has only one ligand

Chemokines are small proteins that act by combining with their cell surface receptors. They take active roles in numerous pathological states and biological processes, including immune response [25], tissue injury [26], cardiovascular diseases [27], and oncogenesis [28]. To date, more than 20 corresponding human chemokine receptors have been identified. Chemokines can be categorized by the position of the conserved cysteine residues [13, 29] into four different groups: C, CC, CXC, and CX3C. A chemokine receptor comprises seven-transmembrane domains, all with three extracellular and three intracellular loops. One of the intracellular loops is coupled with heterotrimeric G-proteins, capable upon binding of the ligand and receptor of triggering a cascade of signal transduction events [29–31]. Receptor nomenclature typically follows that of the chemokines, i.e. CC chemokines bind to CC chemokine receptors, CXC ligands bind to CXC receptors, with a high degree of redundancy in the chemokine family as multiple chemokines bind to the same receptor [29, 32] (Fig. 2).

Tumor cells have the potential to sabotage the chemokine system, in which the molecules and their receptors become important regulators of the TME and major players in cancer biology. With the ability to activate certain signaling pathways, chemokine receptors may facilitate tumor progression at each of the key steps, including proliferation, angiogenesis, immunomodulation, and metastasis [13]. In addition, increasing studies have exhibited the property of chemokines in facilitating information exchanging between cancer cells and TME cells such as endothelial cells and fibroblasts, which in turn promotes the infiltration and activation of immune cells such as neutrophils and tumor-associated macrophages (TAMs) [28].

### MVs and chemokines in TME

**Fig. 3:**
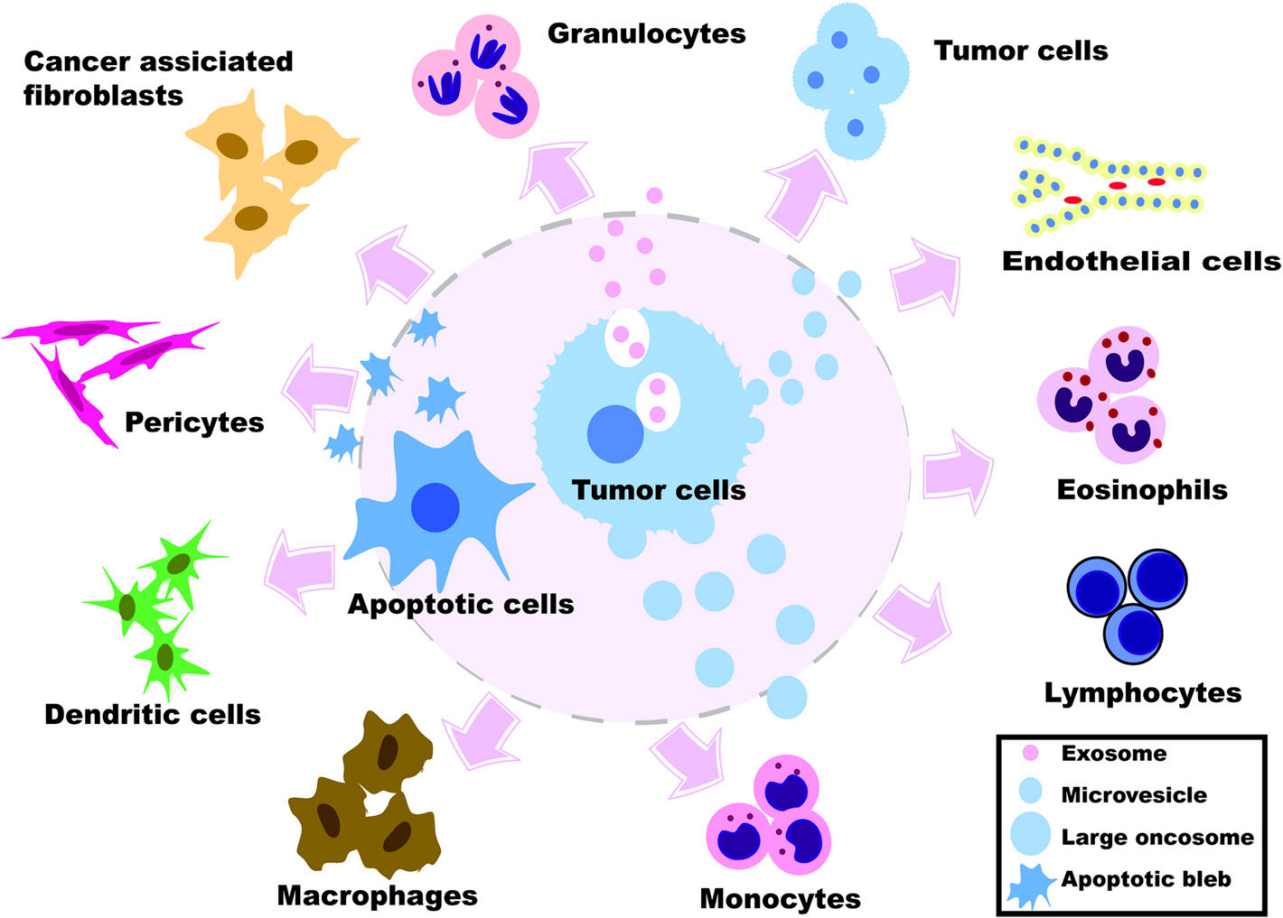
Interaction between cancer cells and different components of the tumor microenvironment by the mediators of EVs

The molecular mechanisms underlying the functional interactions between cancer cells and the TME have been considered the subject of great moment. Historically these interactions are thought to be primarily mediated by signaling molecules such as cytokines and growth factors [33]. TME consists of various cell types, among them are fibroblasts, lymphocytes, inflammatory cells, epithelial cells, endothelial cells, and mesenchymal stem cells [34]. These cells interact with cancer cells and, together they form the intrinsic communication networks that affect several cancer hallmarks (Fig. 3). Studies indicate that such intercellular communications were modulated by various factors, such as growth factors, cytokines, and chemokines. Similar to these molecules, recent advances in cancer biology reveal that MVs also serve as a regulatory agent in such communications [11, 34].

### Peripheral blood system

In recent years, MVs have been described in cancer research as tumor-derived microvesicles (TMVs) [35]. Several reports documented that TMVs induce chemotaxis of leukocytes. Vesicles shed by cell lines of non-small-cell lung carcinoma, pancreatic adenocarcinoma, and colorectal adenocarcinoma stimulated chemotaxis of granulocytes, lymphocytes, and monocytes in vitro [36]. While in the plasma of hemophilia A patients, higher levels of MVs derived from endothelial cells, neutrophils, T lymphocytes, erythrocytes, and platelets were observed after exposure to exogenous FVIII, with distinct immunological profiles [37]. Human eosinophils could secrete cytokines, chemokines and cationic proteins, trafficking, and releasing them for roles in inflammation and other immune responses. When eosinophils are activated immediately after isolation and dissected by transmission electron microscopy, EVs are identified as MVs outwardly shedding off the plasma membrane. Both chemoattractant protein-11 (CCL11) and tumor necrosis factor-α (TNF-α) induce significant increase of MVs compared with non-activated cells [38].

### Macrophage-monocyte system

TMVs carry several proteins and mRNA of tumor cells and can transfer some of them to monocytes. It is found that TMVs could activate monocytes, as evident by increased human leukocyte antigen-DR isotype (HLA-DR) expression, induce production of reactive oxygen intermediates (ROI), and mRNA accumulation and protein secretion of TNF, interleukin (IL)-10, IL-12p40 [36]. Furthermore, TMVs can exert anti-apoptotic effect on monocytes and activate serine/threonine kinase (AKT) by transferring CCR6 and CD44v7/8 to monocytes, altering immunologic phenotype and biological activity of the recipients [39]. TMVs induce expression of IL-8 (CXCL8), monocyte chemoattractant protein-1 (CCL2), macrophage inflammatory protein-1α (CCL3) and major intrinsic protein of lens fiber-1β (MIP-1β) (CCL4), and regulate on activation normal T-cells expressed and secreted CCL5 chemokines and accumulation of their mRNA in monocytes. Moreover, TMVs enhance angiogenesis in non-obese diabetic/severe combined immunodeficiency (NOD-SCID) mice by delivering chemokines and via stimulation of monocytes [40]. Monocytes are direct precursors of hematopoietic stem cell-derived macrophages. After their recruitment into the tumor tissue, they can differentiate into tumor-associated macrophages, and support tumor initiation, local progression, and distant metastasis [41]. It is becoming clear that macrophages, like other members of the myeloid family, are incredible heterogeneous and depending on tumor biology, different subpopulations of tumor associated macrophages may differ considerably in terms of function and phenotype [42]. In B16-F0 melanoma or EL-4 lymphoma cell lines, tumor cell-derived exosomes (TE) enhance the ability of mesenchymal stromal cells (MSCs) to promote macrophage infiltration. Ablation of macrophages by clodronate liposome administration reverses the tumor-promoting effect. In this process, TE-MSCs produce a large amount of CCR2 ligands, CCL2 and CCL7, which are responsible for macrophage recruitment [43].

### Stromal cells

Increasing evidence has disclosed that stromal cells in the TME are also fundamental in tumor progression [44, 45]. Apart from extracellular matrix (ECM), TME includes non-malignant stromal cells surrounding the tumor cells, including fibroblasts, adipocytes, endothelial cells and inflammatory immune cells. The interplay between tumor cells and TME has been increasingly recognized as a principle determinant of malignancy. The stromal elements secrete chemokines functioning in a paracrine manner, which could induce ECM remodeling and enhance cancer proliferation and invasion. For example, we recently found that overexpression of the chemokines CXCL14 and CCL17 in mammary fibroblasts could enhance proliferation, migration, invasion of breast cancer epithelial cells, and contribute to chemo-resistance and disease relapse [45]. Chemokines of the CXCL family are present in the pancreatic TME and play a vital role in regulating PC progression. Most members of the chemokine family, including CXCL1, CXCL2, CXCL5, CXCL9, CXCL10 and CXCL13, where they are secreted by cancer or stromal cells, such as cancer-associated fibroblasts (CAFs) and dendritic cells (DCs). Most of these ligands have been reported to promote chemoresistance, immunosuppression, tumor proliferation and metastasis [46]. In the HIC1-deleted breast cancer cells, CXCL14 bound to its novel cognate receptor GPR85 on CAFs in the TME and was responsible for activating these fibroblasts via the extracellular regulated MAP kinase1/2 (ERK1/2), AKT, and neddylation pathways, promoting cancer progression via the induction of the epithelial-mesenchymal transition (EMT) by the CCL17/CCR4 axis [45].

Large oncosome-induced migration of CAFs can be potentiated by EVs derived from tumor cells in which miR-1227 has been overexpressed. Interestingly, this forced expression of the miRNA intracellularly results in a 3-fold change in large oncosomes in comparison to exosomes [47]. Large oncosomes can also potently stimulate expression of metastasis-associated factors, such as brain-derived neurotrophic factor (BDNF), CXCL12 and osteopontin, in stromal cells [24]. Normal T-cells secrete CCL5, which stimulates the externalization of S100 calcium binding protein A4 (S100A4) via MVs shedded from the plasma membrane of tumor and stromal cells. In wild type and S100A4-deficient mouse models, tumor cell-derived CCL5 on S100A4 release into blood circulation ultimately increases the metastatic burden in mice [48]. EVs produced by the highly metastatic rat pancreatic adenocarcinoma cell line BSp73AS preferentially target lung fibroblasts and lymph node stromal cells, triggering in these cells the upregulation or de novo expression of several adhesion molecules, chemokines, growth factors, and proteases, thus promoting pre-metastatic niche formation [49].

### Dendritic-T cell system

TMVs are natural cargos for delivering tumor antigens and innate signals to DCs for tumor-specific T cell immunity. TMVs, once entering intestinal lumen, were mainly taken up by ileac intestinal epithelial cells (IECs), where TMVs activated nucleotide binding oligomerization domain containing 2 (NOD2) and its downstream mitogen activated kinase-like protein (MAPK) and nuclear factor-κB (NF-κB), leading to release of chemokines including CCL2, from IECs to attract CD103^+^ CD11c^+^ DCs, leading to subsequent antitumor T cell responses [50]. In the cohort of previously untreated hemophilia A patients, immunological profiles were distinct, higher levels of IL8, IL6, IL4, IL10, IL2, IL17A, and lower levels of CXCL10 and CCL2 were observed compared with non-haemophiliac cohorts. Also, higher levels of MVs derived from endothelial cells, neutrophils, T lymphocytes, erythrocytes, and platelets were observed [37]. Few leukemia-associated antigens (LAA) are characterized for acute myeloid leukemia (AML), apoptotic tumor cells constitute an attractive LAA source for personalized DC-based vaccines. DCs preferably ingest apoptotic blebs (MVs that require additional isolation steps) and are superior in migrating toward CCL19. Co-culturing bleb-loaded DCs with T cells led to an increased CD4^+^ T cell proliferation and increased interferon gamma (IFNγ) production by allogeneic T cells. Superior ingestion efficiency and migration, combined with favorable T cell cytokine release and CD8^+^ T cell priming ability and avidity, point to blebs as the preferred component of apoptotic leukemic cells for LAA loading of DC for the immunotherapy of AML [51]. Karin et al. demonstrated that CXCL10 acted on CD4^+^ and CD8^+^ T cells to enhance anti-tumor immunity, blocking the CCR8-CCL1 interaction, alone or combined with other immune checkpoint inhibitors, as an approach to treat malignant diseases [13]. Heat stressed tumor cells produce chemokine-enriched exosomes which are termed HS-TEX, which chemoattract and activate DCs and T cells more potently than conventional tumor-derived exosomes do. The enriched chemokines include CCL2, CCL3, CCL4, CCL5, and CCL20, enabling chemotaxis of DCs and T cells both in vitro and in vivo. Intratumoral injection of HS-TEX could induce specific antitumor immune response more efficiently than that by tumor-derived exosomes, inhibiting tumor growth and significantly prolonging survival of tumor-bearing mice. Therefore, heat stress may alter the functional attributes of tumor-derived exosomes, and the resulting HS-TEX may be an efficient tumor vaccine [52].

### TMVs and chemokines in tumor progression

**Fig. 4:**
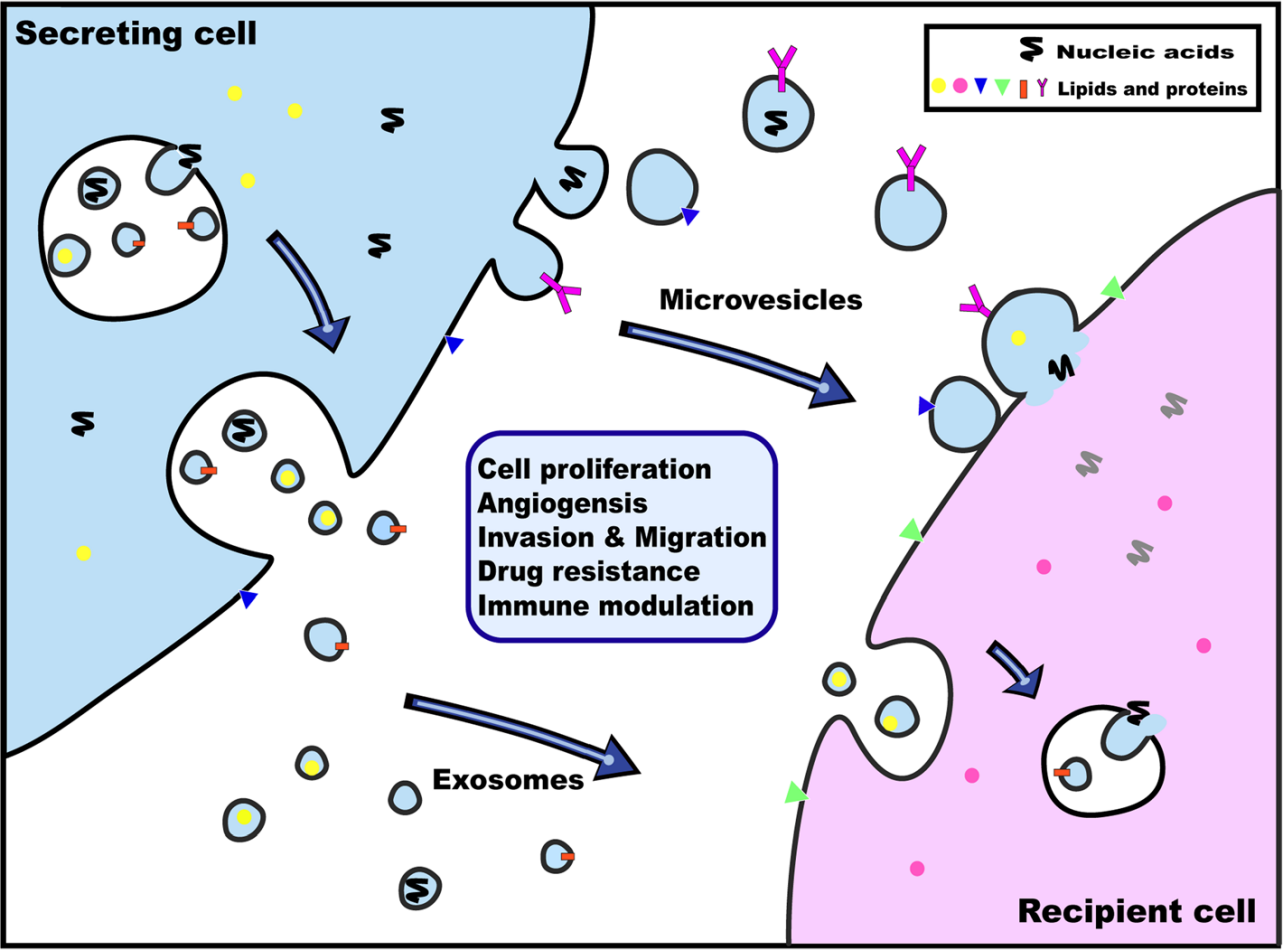
Schematic of molecules and nucleic acids transfer by EVs in the process of cancer progression. Transmembrane and soluble proteins, lipids and nucleic acids are selectively coalesced into the multivesicular endosomes (MVEs) or into MVs shedding from the plasma membrane. MVEs fuse with the plasma membrane to secret exosomes into the extracellular environment. Exosomes and MVs may either be endocytosed by recipient cells or fuse directly with the plasma membrane. Both pathways result in the delivery of proteins, lipids and nucleic acids into the membrane or cytosol of the recipient cell to transfer the information, which potentially influence the progress of cancer development

TMVs have emerged as essential mediators of cancer progression, which alter the metastatic behavior of primary tumors mainly through transport of their bioactive contents including oncogenes, oncoproteins, microRNAs, as well as transcripts of proteins and chemokines involved in angiogenesis or inflammation (Table 1). Moreover, TMVs have been shown to influence distant cellular niches, establishing favorable microenvionmental conditions that support growth of disseminated cancer cells upon their arrival at these pre-metastatic niches (Fig. 4).

**Table 1:** EVs-related specific chemokines in tumor environment

Tumor type	EVs type	Content of chemokines	Role of Evs	References
Breast cancer	MVs	CCL5, CCR6	metastasis, proliferation and cancer cell-induced angiogenesis	[5, 47]
Colorectal adenocarcinoma	MVs	CCR6, CX3CR1, CCL2	antiapoptotic effect on monocytes, AKT kinase activation,proliferation	[38, 49]
Gastric cancer	MVs	CCR6	tumor progression	[52]
Glioblastoma	EVs	CCR8, CCL18	proliferation and drug resistance	[56]
Haemophilia A	MVs	CXCL8, CXCL10, CCL2	immunological profile	[36]
Leukemia	Apoptotic blebs	CCR7, CCL19	immuno modulation	[50]
	Exsomoes	CCL3, CCL4, CXCR4	cell survival and drug resistance	[74]
	MVs	CXCR4, CCL12	cell survival	[68]
Lung carcinoma	MVs	CCR6, CX3CR1, CXCR4	metastasis, antiapoptotic effect on monocytes, AKT kinase activation	[38, 58]
	Exsomoes	CCL2, CCL3, CCL4, CCL5, CCL20	antitumor immune response	[51]
Lymphoma	Exosomes	CCL2, CCL7	macrophage recruitment	[42]
Melanoma	Exosomes	CCL2, CCL3, CCL4, CCL5, CCL7, CCL20	macrophage recruitment, antitumor immune response	[42, 51]
	MVs	CCL2	inhibi ttumor proliferation	[49]
Pancreatic adenocarcinoma	MVs	CCR6, CX3CR1, CCL2, CCL3, CCL4, CCL5, CXCL8	anti-apoptosis, AKT kinase activation, angiogenesis	[38, 39]
Prostate cancer	Oncosomes	CXCL12	oncogenic signaling	[23]

### Proliferation

Cancer cells release significantly larger amount of shedding MVs than their normal counterparts, which are associated with increased invasiveness and disease progression. Genetic alterations are needed to ensure sustained growth and proliferation of cancer cells and MVs facilitates intercellular spread of oncogenes, therefore enabling horizontal transfer of aggressive phenotypes. In gastric cancer patients, the amount of circulating MVs was elevated in all stages compared to normal people and, significantly higher in more advanced disease. MVs of these patients exhibited an increased expression of CCR6 and HER-2/neu on the membrane [53]. CD44H, CD44v6 and CCR6 molecules may play a role in attachment of TMVs to cancer cells, while HER-2 associated with CD24 may be involved in promoting growth of cancer cells. Pre-exposure of cancer cells to these TMVs resulted in enhancement of tumor growth and cancer cell-induced angiogenesis in vivo [5]. Interestingly, this signature presents important overlaps with other microenvironmental stimuli such as B-cell receptor stimulation, CLL/nurse-like cells co-culture or those provided by a lymph node microenvironment. EVs from MSCs of leukemic patients also rescue leukemic cells from spontaneous or drug-induced apoptosis, resulting in a higher migration and also a stronger gene modification [54].

Indolent glioma cells can acquire epidermal growth factor receptor variant III (EGFRvIII), a tumor-specific truncated form of EGFR, from microvesicles released by aggressive glioma cells harboring this variant [55]. Activation of growth promoting MAPK and AKT signaling pathways ensues, resulting in morphological transformation and anchorage-independent growth of the recipient tumor cells. In vitro studies confirmed that transfer of MV-encapsulated EGFRvIII mRNA also stimulated proliferation of glioblastoma cells [56], although it is unclear whether this signaling pathway is active in vivo. Interestingly, the uptake of TMVs is mediated by PS exposed on the surface, which can be blocked using Annexin V or EGFRvIII kinase inhibitors, further confirming the association between phenotypic switch and MVs transfer of oncoproteins or oncogenes. By in vitro and in vivo stem-like glioblastoma models, EVs isolated from glioblastoma-conditioned media with PKH67-label induce a proliferative phenotype in recipient glioblastoma cells. Using fluorescence activated cell-sorting analysis, the percentile of PKH67^+^ cells after incubation showed a sigmoidal log-linear dose-dependent relationship with the amount of PKH67-labelled EVs added. CCR8 acts as an EV receptor on glioblastoma cells and binds to CCL18, which acts as a bridging molecule. CCR8 inhibition caused a strong and consistent reduction in EVs uptake, neutralizes EVs-induced phenotypes in vitro [54].

In addition, MVs are capable of transitioning normal cells in TME into anaplastic cells. Antonyak et al. [57] demonstrated that MVs released by MDA-MB-231 human breast cancer cell line and U87 glioblastoma cell lines contain tissue transglutaminase (tTG), the protein cross-linking enzyme, and FN, the tTG-binding molecule and cross-linking substrate. Microvesicle-mediated transfer of cross-linked FN and tTG to recipient fibroblasts induces their transformation and aberrant proliferation by phosphorylation of focal adhesion kinase (FAK) and ERK kinases and activation of mitogenic signaling pathways.

### Angiogenesis

Angiogenesis, the process involving formation of new blood vessels, is another hallmark of cancer and is of significance in promoting tumor dissemination and migration. Numerous studies have demonstrated that MVs can interact with endothelial cells, therefore stimulating angiogenic responses. TMVs harboring activated EGFR can be taken up by endothelial cells, leading to activation of MAPK and AKT signaling pathways, accompanied by increased expression of endogenous vascular endothelial growth factor (VEGF) and the autocrine activation of VEGF-2, the key receptor for VEGF signaling pathway [58]. In NSCLC, patient-derived circulating TMVs enhance vascular endothelial growth factor receptor 2 (VEGFR2) expression, as well as angiogenesis, nitric-oxide production, and endothelial cell proliferation. The amount of circulating MVs is highly correlated with pro-angiogenic factors at cellular and protein levels. In another study, rats treated with patient-derived circulating MVs exhibit higher microvessel count, more CXCR4^+^ and VEGF^+^ cells, and accelerate pulmonary metastatic hepatocellular carcinoma growth [59]. Endothelial cells that receive CD138 from multiple myeloma derived MVs are significantly stimulated so as to proliferate, secrete IL-6 and VEGF, two key angiogenic factors of myeloma, and form tubes in vitro and in vivo [60].

Aside from carrying bioactive EGFR variants, the aforementioned glioblastoma derived TMVs are loaded with angiogenic proteins, such as fibroblast growth factor (FGF), IL-6, and VEGF, which are capable of stimulating angiogenesis in vitro [57]. By advancing formation of new blood vessels in human brain microvascular endothelial cells, these TMVs were reported to stimulate cancer proliferation, motility, and tube formation in a dose-response manner [61]. Similarly, Hong et al. [62] identified 241 mRNAs, which were enriched in colorectal cancer cell-derived MVs. Treatment of endothelial cells with these MVs resulted in a significant increase in proliferation, which is in line with the results of network analysis. MVs shed from CD105^+^ human renal cancer stem cells confer an activated angiogenic phenotype to normal epithelial cells, stimulate blood vessel formation after in vivo implantation in severe combined immunodeficient (SCID) mice, and enhance risk of developing lung metastases [63]. Besides pro-angiogenic growth factors, proteinases and cytokines, microvesicles may cargo miRNAs to mediate angiogenesis, as is exemplified in gastric cancer [64] and colorectal cancer [65].

### Metastasis

The invasive and migratory properties of tumor cells accumulate when tumor cells grow and evolve [11]. This hallmark of cancer is associated with MV-encapsulating proteases, such as the MMP family. This family of enzymes can degrade ECM and catalyze the proteolysis of the basement membrane, therefore enhancing the mobility of migrating tumor cells and allowing them to enter the circulatory system. In amoeboid-like invasive melanoma cell lines, vesicular soluble N-ethylmaleimide-sensitive factor attachment protein receptor (v-SNARE) and vesicle-associated membrane protein 3 (VAMP3) have been identified as two key regulators for delivery of the cargo molecules to shedding TMVs, such as the membrane-type 1 matrix metalloprotease (MT1-MMP). These TMVs markedly facilitate the maintenance of amoeboid phenotype and allow for cell invasion. VAMP3-shRNA transfected cells are lacking in TMVs that contain MT1-MMP, making them difficult to invade through dense and highly cross-linked matrices such as rat-tail collagen compared to those transfected with scramble shRNA [4]. VMR, CSML100, and CSML0 mouse adenocarcinoma cell lines originated from two independent spontaneous tumors in A/Sn mice, CCL5 stimulates the externalization of S100A4 via TMVs shedding from the plasma membrane of these tumor and stroma cells, which in its turn induces the upregulation of FN in fibroblasts and a number of cytokines in tumor cells including CCL5 [48]. In TMVs ARF, modulation of ARF1 expression dramatically impairs the ability of MDA-MB-231 cells to degrade the extracellular matrix by adjusting MMP9 activity, to inhibit invasiveness and metastasis [66, 67].

In prostate cancer (PCa) cell lines with mesenchymal characteristics (22Rv1/CR-1; Mes-PCa), TMVs were found to promote and maintain mesenchymal features in the recipient epithelium-like prostate cancer cells, modulating androgen receptor signaling and activating transforming growth factor beta (TGF-β) signaling pathway in the meantime. Moreover, these recipient cells which have attained mesenchymal traits exhibited enhanced migratory and invasive potentials, as well as increased resistance to the androgen receptor antagonist enzalutamide [68]. CXCR7 is a chemokine that has been proven responsible for PCa progression. As a direct downstream target of hypermethylated in cancer 1 gene (HIC1), restoring HIC1 expression in PCa cells markedly inhibited proliferation, migration, and invasion and induced the apoptosis in these cells [69]. In vitro and in vivo studies with PCa cell lines suggest that alterations in CXCR7/RDC1, receptor for SDF-1/CXCL12, are associated with enhanced adhesive and invasive activities, regulates the expression of the proangiogenic factors IL-8 or vascular endothelial growth factor, which are likely to participate in the regulation of tumor angiogenesis [70]. SDF-1/CXCL12 and its receptor CXCR4 are implicated in the pathogenesis and prognosis of AML. MVs with NH(2)-terminal truncation of the CXCR4 molecule are capable of transferring the CXCR4 molecule to AML-derived HL-60 cells, enhancing their migration to SDF-1 in vitro and increasing their homing to the bone marrow of irradiated NOD/SCID/beta2m (null) mice. These effects could be reduced by the CXCR4 antagonist AMD3100 [71]. While in epithelial ovarian cancer, expression of SDF-1/CXCL12 and the genes controlling alternative splicing are elevate, leading to an increased formation of SDF-1 variant 1. No changes in CXCR4 and CXCR7 expression level are observed. Elevated plasma SDF-1α level in epithelial ovarian cancer patients is not associated with the presence of tumors and/or metastases, however reflects a general response to the disease [72].

Coagulation proteins play a critical role in numerous aspects of tumor biology. Tissue factor, which is more frequently referred to by hematologists as thromboplastin or Factor III, can be present in TMVs and correlates well with biological processes related to cell aggressiveness, including tumor growth, invasion, and metastasis. It may therefore contribute to the propagation of a tissue factor associated aggressive phenotype among heterogeneous subsets of cells in a breast cancer [73]. Agonist-stimulated platelets require integrin outside-in signaling to efficiently externalize the procoagulant phospholipid PS and release PS-exposed MVs [74].

### Drug resistance

Therapeutic resistance is the major reason for the poor prognosis of malignancies. Cancer progression is a complex process reliant on interactions between the tumor and the TME [15]. Interactions between chronic lymphocytic leukemia (CLL) B cells and the bone marrow (BM) microenvironment involve in multiple steps in the physiopathology of CLL. Exosomes and MVs purified from BM mesenchymal stromal cell were accessed to integrate into CLL B cells. After 24 h cocultivation, an increase in their chemoresistance to several drugs, including fludarabine, ibrutinib, idelalisib and venetoclax, were observed. In terms of B-cell receptor pathway activation, expression of CCL3/4, EGR1/2/3 and MYC increased, leading to cell survival and drug resistance [75]. EVs isolated from glioblastoma-conditioned media promote cell proliferation and resistance to the alkylating agent temozolomide (TMZ). EV-mediated induction of proliferation is dose-dependent, activating the MAPK-ERK pathway, as evidenced by an increase level of phospho-ERK. This phenomenon could be reversed by pharmacological inhibition of CCR8 with the small molecule R243, inhibiting EV uptake by GBM cells, resulting in sensitization of glioblastoma cells to TMZ [54].

Accumulated studies indicate that TMVs are capable of conferring chemotherapy resistance. This can be achieved via transport from drug-resistant cancer cells to their drug-sensitive counterparts of functional plasma membrane transporter proteins including P-glycoprotein (P-gp), breast cancer resistance protein (BCRP) [76], and multidrug resistance-associated protein 1 (MRP1) [77], or resistance-associated miRNAs [78]. Ezrin, a member of the ezrin/radixin/moesin family of proteins linking plasma membrane to cytoskeleton actins, is transported along with the microvesicular cargo and determine P-gp membrane insertion through a cytoskeletal association, as shown in breast cancer cells [79]. It has also been found that drug-sensitive breast cancer and lung cancer cells experience became resistant to cisplatin or paclitaxel treatment after incubation with shed MVs containing inhibitors of apoptosis proteins (IAPs) [80], suggesting that MVs could activate multiple drug resistance pathways irrespective of cancer types.

Another mechanism for microvesicle-induced drug resistance is the direct expulsion of chemotherapeutic agents from cancer cells. Such has been observed in breast cancer, where doxorubicin and small molecules accumulated in membrane domains in which vesicles originated and released in shed MVs [81]. These observations have led to research on inhibition of MV origination and shedding processes in an attempt to reverse drug resistance. In vitro inhibition of microvesiculation with calpain inhibitor calpeptin and siRNAs sensitize prostate cancer cell line PC3 to chemotherapy, resulting in a 20-fold decrease in the concentrations of docetaxel needed to induce the same degree of apoptosis [82]. In contrast, pharmacological inhibition of peptidylarginine deiminases, a family of enzymes associated with deamination of cytoskeletal actins and vesicle formation significantly reduce microvesicle release and increased the sensitivity of PC3 cell lines to methotrexate treatment [83].

Despite the fact that MVs play important roles in facilitating tumor drug resistance formation, recent studies have focused on utilizing MVs to develop novel approaches to reverse drug resistance. Ma et al. [84] demonstrated that drug-resistant tumor-repopulating cells derived from patients with lung cancer preferentially took up MVs containing cisplatin, which led to reversal of drug resistance and apoptosis of cancer cells. A phase I/II clinical trial investigating the effect of peritoneal perfusion of autologous erythrocyte-derived MVs containing methotrexate on malignant ascites has been registered in the year 2017 and is currently recruiting (NCT03230708).

### Immunomodulation

Cancer immunotherapy, which takes advantage of innate immune response against tumor, has recently brought paradigm shift to cancer treatment. The key concept in immunotherapy is to present cancer-specific immunogens and initiate T cell-mediated cancer immunity. It is for this reason that MVs, which are capable of conveying bioactive molecules and biological information, have received renewed attention. There is complex cross-talk among cancer cells, tumor microenvironment, and the immune system, as evident by the conflicting observations of effects of TMVs. On the one hand, it has been reported that TMVs are more immunogenic than soluble antigens in mouse models [85] as well as human cancer cells [86]. On the other, microvesicle signalling can enhance immunosuppressive characteristics of tumor cells, contributing to escape of immune surveillance and cancer metastasis. Mesenchymal stem cell-derived EMVs, with their capacity to migrate towards inflammatory areas including solid tumors, have been used to carry tumor RNA (RNA-lipoplexes) and provoke a strong anti-tumor immune response mediated by cytotoxic CD8^+^. MVs and exosome-mimetic nanovesicles delivery of siRNA or chemotherapeutic drugs that target tumors using peptide ligands for cognate receptors on the tumor cells are discussed [87]. In mice models, TMVs by oral vaccination route effectively access and activate mucosal epithelium, resulting in subsequent antitumor T cell responses. Oral vaccination of TMVs inhibited the growth of B16 melanoma and CT26 colon cancer, which required both T cell and DC activation. Taken up by IEC in intestinal lumen, TMVs activated NOD2 and its downstream MAPK and NF-κB, leading to chemokine releasing, including CCL2, from IECs to attract CD103^+^/CD11c + DCs [50]. Maus et al. [88] showed that melanoma-derived MVs compromised the maturation process of DCs, the latter exhibiting significantly decreased expression of CD83, CD86, migratory chemokines MIP-1, and Th1 polarizing chemokines Flt3L and IL15. Alternatively, this immunosuppressive effect of MVs can be achieved by promoting the differentiation of myeloid cells toward myeloid-derived suppressor cells [89], which are known to counteract anti-tumor immunity. Compared to apoptotic AML cell remnants, apoptotic blebs derived from apoptotic AML cells are preferably ingested by DCs and induce their lymph node migration capacity. Co-culturing these bleb-loaded DCs with T cells led to an increased production of IFNγ compared to co-culture with unloaded or apoptotic cell remnant-loaded DCs. Considering that LAAs are scarcely characterized for AML, and that loading DCs directly with apoptotic AML cell remnants may compromise DC functions, apoptotic blebs provide an attractive and potent LAA source for developing personalized DC-based vaccines against AML [51]. Studies by the Rughetti group [90, 91] revealed that microvesicle-mediated antigen transfer to DCs is of crucial importance for cross-presentation of tumor-glycosylated antigens. In particular, mucine 1 (MUC1), one of the most relevant glycoproteins associated with carcinogenesis, was cross-processed and presented to antigen-specific CD8^+^ T cells when carried by MVs, while the internalized soluble form of MUC1 was retained in the endolysomal/HLA-II compartment and did not activate any T cell response. They further proposed that the controversial roles of MVs in modulating immunity are dependent upon the stage of tumor progression.

DC-derived exosomes contain series of costimulatory molecules including B7–1 (CD80), B7–2 (CD86), programmed death 1-ligand (PD-L1) and PD-L2. Rather than PD-L1 and PD-L2, therapeutic effects of IL-10 treated DC and exosomes required both B7–1 and B7–2, which play a critical role in immunosuppressive functions of both DC and exosomes, giving the growing interest in exosomes for therapeutic applications [92]. In glioblastoma, PD-L1 was expressed on the surface of some glioblastoma-derived EVs, with the potential to directly bind to programmed death-1 (PD1). These EVs block T cell activation and proliferation in response to T cell receptor stimulation. Blocking PD1 pathway significantly reversed the EV-mediated blockade of T cell activation but only when PD-L1 was present on EVs. When glioblastoma PD-L1 was up-regulated by IFN-γ, EVs also showed some PD-L1 dependent inhibition of T cell activation [93]. HER2-positive breast cancer cells with stable over-expressing Neuromedin U and their released EVs have increased amounts of the immunosuppressive cytokine TGFβ1 and the lymphocyte activation inhibitor PD-L1, show enhanced resistance to antibody-dependent cell cytotoxicity mediated by trastuzumab, indicating a role of Neuromedin U in enhancing immune evasion [94]. While in malignant glioma, monocytes from naïve patient peripheral blood treatmented with glioma-derived exosomes fail to induce monocytic PD-L1 expression or alter the activation of cytotoxic T-cells, but promote immunosuppressive HLA-DR low monocytic phenotypes [95].

Probably the most promising future for therapeutic use of MVs in cancer immunotherapy is to be administered as vaccines. In their study, Zhang et al. [96] immunized mice with extracellular vesicles isolated from different cancer cell lines, and as a result, 50% of the microparticle-immunized mice remained tumor-free after injected tumor challenges. They further discovered that tumor-derived microvesicles confer DNA fragments to DCs, leading to type I IFN production through the cGAS/STING-mediated DNA-sensing pathway. Type I IFN, in its turn, stimulate DC’s maturation and antigen-presenting capabilities. Notably, Zhang et al. reported a much lower 12.5% tumor-free rate of exosome-immunized mice after the tumor challenges. This suggests that, although the present development of extracellular vesicle-based vaccines is largely focusing on exosomal vaccines, microparticle-based vaccines appear to be more immunogenic.

Taken together, these studies highlight the potential clinical applicability of microvesicle-based vaccines in cancer immunotherapy. In future, these vaccines are expected to be administered alongside immune checkpoint inhibitors, the currently well-established immunotherapeutic approach, to further augment anti-tumor immunity.

## Conclusions

As evidence has been shown from the literature, MVs are extensively studied and greatly contribute to the pathogenesis of multiple cancer types. With the growing understanding of the biology and biogenesis of MVs in cancer pathophysiology, MV research has been spawning much excitement in the past decade. Of particular interest for the current discussion is the intercellular communication between cancer cells and stromal cells in TME, which frequently involves bidirectional transfer of encapsulated chemokines. Presently available studies have looked at TMVs using in vitro cell lines or in vivo animal models, and revealed the importance of MVs as key mediators of cancer growth, proliferation, apoptosis, angiogenesis, coagulation and metastasis, proposing a paradigm shift of using TMVs as diagnostic or prognostic biomarkers. Moreover, TMVs have been shown to contribute to chemo-resistance and immunomodulation of cancer cells, shedding light on the clinical application of TMV-based or TMV-targeted therapeutic interventions to augment the efficacy of chemotherapy or immunotherapy. Nevertheless, current understanding of the TMVs and TMV-related chemokines has only scratched the surface. In order to demonstrate the authentic physiological functions of MVs in vivo, it is imperative to design animal models in which release and uptake of chemokine-containing MVs can be specifically monitored and interfered. With the mechanism of how MVs mediate intercellular communication becomes increasingly appreciated, MVs are expected to reshape our view towards cancer biology, become important component in laboratory research and elucidate novel therapeutic strategies for various cancer types.

## Abbreviations

AKT: Serine/threonine kinase
AML: Acute myeloid leukemia
ARF6: ADP-ribosylation factor 6
BCRP: Breast cancer resistance protein
BDNF: Brain-derived neurotrophic factor BM Bone marrow
CAF: Cancer-associated fibroblast
CD: Cluster of differentiation
CHAMP3: Charged multivesicular body protein 3
CLL: Chronic lymphocytic leukemia
DC: Dendritic cell
ECM: Extracellular matrix
EGFR: Epidermal growth factor receptor
EMT: Epithelial-mesenchymal transition
ERK: Extracellular regulated MAP kinase
ES: Embryonic stem
EV: Extracellular vesicles
FAK: Focal adhesion kinase
FN: Fibronectin
HIC-1: Hypermethylated in cancer 1
HLA-DR: Human leukocyte antigen-DR isotype
HS-TEX: Heat-stressed tumor cells
IAP: Inhibitors of apoptosis protein
IEC: Intestinal epithelial cell
IFNγ: Interferon gamma
IL: Interleukin
LAA: Leukemia-associated antigen
MAPK: Mitogen activated kinase-like protein
MIP-1β: Major intrinsic protein of lens fiber-1β
MMP: Matrix metalloproteinase
MRP1: Multidrug resistance-associated protein 1
NF-κB: Nuclear factor-κB
NOD2: Nucleotide binding oligomerization domain containing 2
MSC: Mesenchymal stromal cell
MT1-MMP: Membrane-type 1 matrix metalloprotease
MUC1: Mucine 1
MVE: Multivesicular endosome
MV: Microvesicle
PD1: Programmed death 1
PD-L1: Programmed death 1-ligand
P-gp: P-glycoprotein
PS: Phosphatidylserine
ROI: reactive oxygen intermediates
S100A4: S100 calcium binding protein A4
SCID: Severe combined immunodeficient
TAM: Tumor-associated macrophages
TE: Tumor cell-derived exosomes
TGFβ: Transforming growth factor beta
TNF-α: Tumor necrosis factor-α
TME: Tumor microenvironment
TMV: Tumor-derived microvesicles
TMZ: Temozolomide
tTG: Tissue transglutaminase
PCa: Prostate cancer
uPA: Urokinase plasminogen activator
VAMP-3: Vesicle-associated membrane protein 3
VEGF: Vascular epithelium growth factor
VEGFR: Vascular endothelial growth factor receptor
v-SNARE: Vesicular soluble N-ethylmaleimide-sensitive factor attachment protein receptor
